# Data Processing and Text Mining Technologies on Electronic Medical Records: A Review

**DOI:** 10.1155/2018/4302425

**Published:** 2018-04-08

**Authors:** Wencheng Sun, Zhiping Cai, Yangyang Li, Fang Liu, Shengqun Fang, Guoyan Wang

**Affiliations:** ^1^College of Computer, National University of Defense Technology, Changsha 410073, China; ^2^Innovation Center, China Academy of Electronics and Information Technology, Beijing 100041, China; ^3^School of Data and Computer Science, Sun Yat-sen University, Guangzhou 510006, China; ^4^Xuzhou University of Technology, Xuzhou 221002, China

## Abstract

Currently, medical institutes generally use EMR to record patient's condition, including diagnostic information, procedures performed, and treatment results. EMR has been recognized as a valuable resource for large-scale analysis. However, EMR has the characteristics of diversity, incompleteness, redundancy, and privacy, which make it difficult to carry out data mining and analysis directly. Therefore, it is necessary to preprocess the source data in order to improve data quality and improve the data mining results. Different types of data require different processing technologies. Most structured data commonly needs classic preprocessing technologies, including data cleansing, data integration, data transformation, and data reduction. For semistructured or unstructured data, such as medical text, containing more health information, it requires more complex and challenging processing methods. The task of information extraction for medical texts mainly includes NER (named-entity recognition) and RE (relation extraction). This paper focuses on the process of EMR processing and emphatically analyzes the key techniques. In addition, we make an in-depth study on the applications developed based on text mining together with the open challenges and research issues for future work.

## 1. Introduction

With the development of information technology and HIS (hospital information system), EMR has also been popularized. EMR (electronic medical record) or EHR (electronic health record), which medical staff uses to record texts, symbols, charts, graphics, data, and other digital information generated by HIS, refers to medical records, which could be stored, managed, transmitted, and reproduced efficiently. With the tremendous growth of the adoption of EMR, various sources of clinical information (including demographics, diagnostic history, medications, laboratory test results, and vital signs) are becoming available, which has established EMR as a treasure trove for large-scale analysis of health data.

Data in EMR can be divided into three kinds: structured data, semistructured data, and unstructured data [1]. Structured data, which is generally stored in fixed-mode databases, contains basic information (such as birth data and nationality), drugs taken, allergies, and vital signs (such as height, weight, blood pressure, and blood type,). Semistructured data usually has the flow chart format, similar to RDF (resource description files), including name, value, and time-stamp.

Unstructured text is one kind of narrative data, including clinical notes, surgical records, discharge records, radiology reports, and pathology reports. Unstructured texts store a lot of valuable medical information but lack common structural frameworks, and there are many errors, such as improper grammatical use, spelling errors, local dialects, and semantic ambiguities, which increase the complexity of data processing and analysis.

This paper proceeds as follows. In Section 2, we introduce the general procedure of EMR data preprocessing and the popular Chinese word segmentation systems. In Section 3, we discuss several classical techniques in EMR data preprocessing. In Section 4, we discuss the information extraction of EMR based on text mining and research status of named-entity recognition and relation extraction. In Section 5, we introduce three main areas of application developed based on text mining. Finally, we analyze several aspects that need to be paid more attention to and conclude the survey in Section 6.

## 2. General Procedure of EMR Data Processing

**Figure 1:**
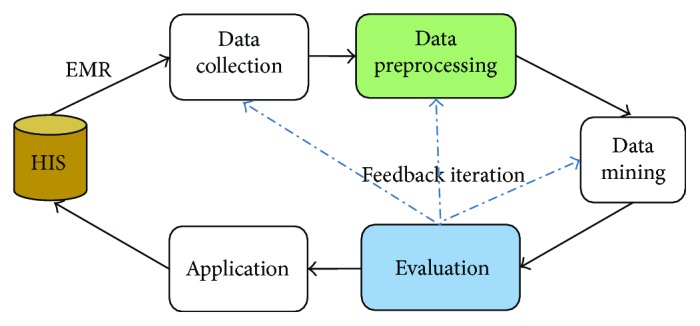
EMR data processing flow.

As shown in Figure 1, the general process of EMR data processing includes data collection [2–4], data preprocessing, data mining, evaluation, and knowledge application.

Data collection is mainly carried out by the government and professional medical institutes. Knowledge application, which is not only the goal of data processing but also the driving force, is more involved in medical management and treatment program disposal. There are many data mining technologies, such as classification, clustering, association rules, and regression. It is only after careful consideration of the dataset that we can make a choice and establish a predictive model. Evaluation means that we need to arrange some tests for the model built, in order to grasp its performance. Besides, the patterns and knowledge excavated also need to be analyzed and optimized. Therefore, the data processing is a process of interactive iteration and requires continuous corrective feedback. Only in this way can we get a relatively better knowledge model.

However, it must be pointed out that the data complexity of EMR has made it difficult to analyze data directly, which needs to be effectively preprocessed. High-quality data is more likely to bring high-quality results. According to statistics, in the entire data processing process, the workload of the preprocessing stage is more than 60%. This paper would sum up those diverse preprocessing technologies and analyze the future trends of Chinese EMR.

The characteristics of unstructured medical texts should be taken into account. The common method is to partition the unstructured data reasonably (also called word segmentation) and then store the segmentation results into a standard database. There are many kinds of word segmentation tools both for Chinese and English texts, but the effect of English word segmentation is generally better. This is because there is no spacer between Chinese words, but there is a space, used as the spacer, between English words.

For the moment, the popular Chinese word segmentation systems include ICTCLAS, Ansj (ICTCLASs java implementation), HTTPCWS, SCWS, PhpanAlysis, MMSEG4J, PanGu Segment, IKAnalyzer, imdict-chinese-analyzer, and LTP-cloud. In the field of Chinese word segmentation, the Institute of Computer Research of the Chinese Academy of Sciences started earlier and achieved more research findings.

## 3. Classical Data Preprocessing on EMR

**Figure 2:**
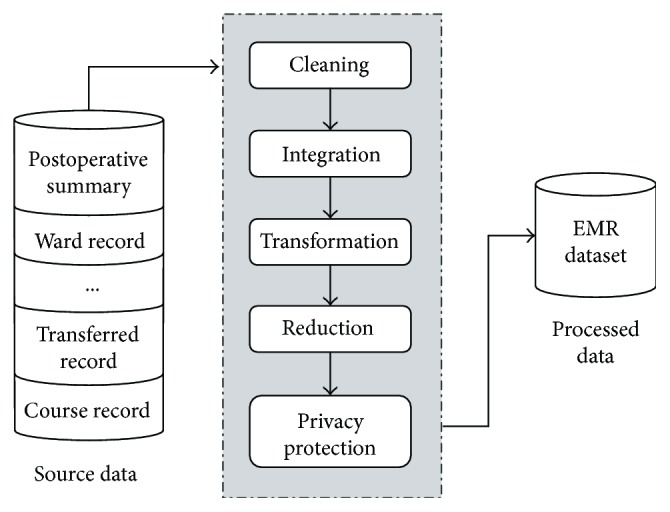
Process of data preprocessing on EMR mining.

Usually, the EMR database is composed of a variety of heterogeneous data sources, and the data retrieved from EMR database is diverse, incomplete, and redundant, which will affect the final mining result to a great extent. Therefore, the EMR data must be preprocessed to ensure that the EMR data is accurate, complete and consistent, and has protected privacy. The process of data preprocessing includes data cleansing, data integration, data transformation, data reduction, and privacy protection, as shown in Figure 2. It should be pointed out that the strategies adopted at each stage of the preprocessing are related. Accordingly, the preprocessing methods should be chosen reasonably, especially for medical data.

### 3.1. Data Cleansing

The EMR data, which is incomplete, noisy, and inconsistent, should be improved by filling defaults, smoothing noise, and correcting data inconsistency.

#### 3.1.1. Data Cleansing

When gathering EMR data, some data attributes may be lost due to manual errors and system failure. For default data, there are several ways around this. We could ignore missing data, manually fill default values, use attribute averages, fill defaults with the most likely values, or retrieve other data sources.

When the missing value has great influence on the processing process, the missing data is usually ignored. For example, when extracting patient information, if the operation name is lost, the data should be ignored; but if the bed number information is lost, the data cannot be ignored. In the case where the dataset is relatively small, the defaults could be manually filled. However, when dealing with larger sets with more defaults, it does not work. In addition, this method is time-consuming and costly, so it is generally not applied. In the case where the data distribution is uniform and the cost budget is not much, the defaults could be filled with the attribute averages. Besides, for default data, machine learning methods can be utilized to determine the optimum value, including regression, Bayesian formal methods, and decision tree induction. Although the prediction may show a relatively great deviation in extreme cases, these methods are still able to better deal with data defaults. Furthermore, when the missing data attribute exists in other data source, the data source should be retrieved.

#### 3.1.2. Noise Processing

Noise refers to an abnormal attribute value in a data source, also known as an illegal value. For example, the patient has a temperature of 27.8 degrees centigrade, a pH of 3.26 (the normal range is 5.00–9.00) or a specific gravity of urine (SG) of 1.96 (the normal range is 1.01–1.03). The processing of noise data includes binning, regression, outlier analysis, and retrieval of other data sources.

The binning methods smooth the ordered data values by examining the values around the data. The key of binning methods is the size of the subbox. The regression method is to modify the noise value by setting up the function model that fits the data attribute value. Outlier analysis is to build clusters by the clustering method. The attributes of data points within the same cluster are similar, but the attribute values of data points between different clusters have a large deviation.

#### 3.1.3. Inconsistent Data Processing

There may be inconsistencies in different sources or homologous data, such as inconsistencies in measurement units and recorded values. The inconsistency of data can be corrected by analyzing the correlation between the data and retrieving the different data sources.

### 3.2. Data Integration

At the data integration stage, the data stored in different data sources needs to be consolidated, and the challenge is to deal with heterogeneous data and its redundancy. Through data integration, the accuracy and speed of data mining can be improved.

#### 3.2.1. Heterogeneous Data Processing

EMR data may be collected from multiple EMR systems, and the different data sources will naturally lead to the heterogeneous problems. Heterogeneous problems are mainly represented by inconsistencies in data attributes, such as attribute names and measurement unit. For example, the expression of specific gravity of urine, which can be SG or specific gravity, and the measurement unit of triglycerides can be mmole/L, but sometimes mg/dl.

#### 3.2.2. Redundant Data Processing

In a nutshell, if an attribute can be derived from other attributes then the attribute is redundant, which should be cleaned up. Redundancy is mainly reflected in the repeated records of data attributes or inconsistencies in the way of attribute expression. For example, when a patient needs to be transferred to other hospitals for treatment, some inspections would be repeated in the latter hospital, which results in repeated medical records that are redundant.

Most redundant data can be detected by correlation analysis. When given two attributes, we can analyze how much one attribute has relevance to the other using the existing data. For nominal data, the commonly used analysis method is chi-square test.

### 3.3. Data Reduction

On the premise of maintaining data integrity, data reduction can reduce the dataset size, which can support data mining in terms of convenience and efficiency. In China, a large amount of EMR would be generated every day. Given the circumstances, data reduction is quite necessary to perform. Data reduction methods include dimension reduction, quantity reduction, and data compression. Among them, dimension reduction, which is easier to fulfill with a better effect, is a relatively popular method.

Dimension reduction method generally controls the size of the dataset by reducing the number of random variables or attributes. Dimension reduction method includes wavelet transform and principal component analysis, which project the source data into a smaller dataset. Attribute subset selection is also a method of dimension reduction, which reduces the size of a dataset by detecting and deleting irrelevant, weak-correlated, or redundant attributes or dimensions.

### 3.4. Data Transformation

Data transformation refers to the conversion of dataset into a unified form suitable for data mining. Data transformation methods include smoothing noise, data aggregation, and data normalization. According to the direction and target of data mining, data transformation method filters and summarizes EMR data. Data analysis can be more efficient by having a directional, purposeful data aggregation.

In order to avoid the dependency of the data attributes on the measurement units, data should be normalized to make the data fall into smaller common spaces, such as [0,10], which is more readable. There are three forms of normalization, including min–max normalization, zero-mean normalization, and fractional scale normalization. For neural network algorithms or classification algorithms based on distance measures (such as nearest neighbor classification), the normalization method works better.

### 3.5. Privacy Protection

Compared with paper medical records, the application of EMR has greatly promoted the development of medical care, but it has also brought a lot of security problems. EMRs contain sensitive information about the patient's privacy, and it can be very serious if they are obtained by lawbreakers. In 2011, the Chinese government issued the “Functional Norms of Electronic Medical Records System (For Trial)” and stressed that the EMR system should realize the function of protecting patient's private information.

There are two main ways to protect the privacy in EMR, including data protection protocols and access control methods. The technical issues involved include data encryption, privacy anonymity processing [5], and access control. In addition, with the emphasis on privacy and sensitive information, the privacy protection system for EMR systems is also gradually established. In addition, with the popularity of SDN technology, the security and related issues in this area have also attracted much attention [6–8].

## 4. Information Extraction of EMR Based on Text Mining

Text mining, also known as text data mining, is designed to acquire implicit knowledge that is hidden in the unstructured text. A wealth of valuable information can be discovered from biomedical texts, such as identifying adverse drug reaction or making early judgments about the patient's symptoms.

**Figure 3:**
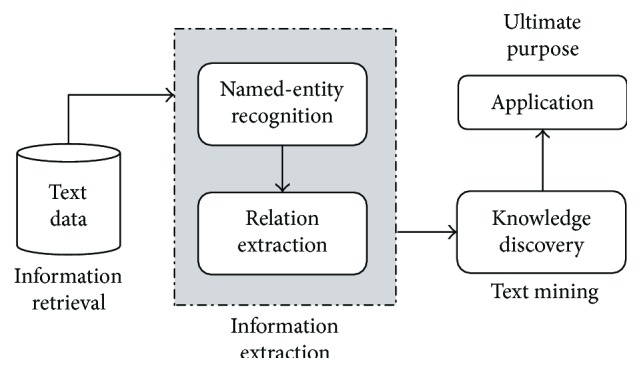
Progress of text mining.

As shown in Figure 3, the text mining process is usually composed of four stages: information retrieval, information extraction, knowledge discovery, and knowledge application. The process of text mining is similar to that of classical data processing. Information retrieval, intended to obtain the desired texts, is similar to data collection. Information extraction is used to extract predefined information, that is, preprocessing of the collected data. Knowledge discovery helps us to extract new knowledge from the text. Knowledge application is the ultimate goal of applying the unknown facts inferred from texts to practice. Medical text mining is mainly for the semistructured and unstructured texts in the professional medical field, so the traditional preprocessing technology cannot be applied directly. The main strategy is to convert semistructured and unstructured texts into computer-readable-structured data by means of information extraction and natural language processing (NLP) technologies. In this process, the key technologies involved include named-entity recognition (NER) and relation extraction (RE).

### 4.1. Named-Entity Recognition Technology

In 1995, the NER task, which refers to the process of identifying particular types of names or symbols in document collections, was introduced for the first time at the MUC-6 (Message Understanding Conference) [9]. In the field of EMR, the NER method is used to identify medical entities that have specific significance for the treatment, such as disease names, symptoms, and drug names. Named-entity recognition, the basic project of text mining, is an important part of information extraction. NER has two steps, entity boundary identification and entity class determination. In the medical field, NER encounters many obstacles, such as the doctor's writing styles (typos and grammar mistakes), different writing forms of medical terms (such as epilepsy and atrophy, which refer to the same disease), and ambiguity in term abbreviations (such as PC, which can refer to prostate cancer, phosphatidylcholine, or personal computer). In addition, some medical terms are composed of phrases or compounds or modified, which is particularly prominent in Chinese texts. All of these issues will reduce the effect of entity recognition.

Three evaluation indexes serve NER, that is, precision rate (P), recall rate (R), and F-score, as shown in the following formulas. F-score, the harmonic average of the precision and recall rate, is a comprehensive evaluation of the test results. When the F-score is higher, the experimental results are better.

(1) $$\begin{array}{c} P=\frac{\text{the }number\text{ of }entities\;identified\;correctly}{\text{the }number\text{ of }entities\;identified},\\ R=\frac{\text{the }number\text{ of }entities\;identified\;correctly}{\text{the }number\text{ of }entities\;present\text{ in the }test\;set},\\ F‐score=\frac{P*R*2}{P+R}. \end{array}$$

Identification of clinical events (e.g., problems, tests, and treatments) and associated temporal expressions (e.g., dates and times) is a key task in extracting and managing data from electronic health records. In the medical field, NER methods can be divided into three types: the rule-based approach, the dictionary-based approach, and the machine learning approach.

#### 4.1.1. The Rule-Based NER Approach

Rule-based approaches need to identify the rules of the named entity from medical texts, and the identified rules are valid only in specific datasets, otherwise invalid [10]. In addition, the rule-based approach and dictionary-based approach require medical expert assistance to construct rule templates and dictionaries manually.

Savova et al. [11] presented an open-source natural language processing system for information extraction from electronic medical record clinical free-text. The clinical Text Analysis and Knowledge Extraction System (cTAKES) they proposed is a modular system of pipelined components combining rule-based and machine learning techniques, released open-source. The NER performance is reported in terms of F-score, while the assignment of attribute values for each discovered named entity in terms of accuracy because each attribute is assigned a label. The best results achieve an F-score of 0.715 for exact matches and 0.824 for overlapping matches.

Tracking the progression of risk factors in EMRs over time may support medical personnel in making clinical decisions, as well as facilitate data modeling and biomedical research. However, this method may not always provide the correct associations. In light of this, Chang et al. [12] introduced a context-aware approach to assign the time attributes of the recognized risk factors by reconstructing contexts that contain more reliable temporal expressions. The evaluation results on the i2b2 test set demonstrate the efficacy of the proposed approach, which achieved an F-score of 0.897.

Hanisch et al. [13] constructed the ProMiner system by a rule-based approach and a preprocessed synonym dictionary, to identify potential name occurrences in the biomedical text and associate protein and gene database identifiers with the detected matches. In blind predictions, the system achieves an *F*-measure of approximately 0.8 for the organisms mouse and fly and about 0.9 for the organism yeast.

#### 4.1.2. The Dictionary-Based NER Approach

The dictionary-based approach, which is well suited for accurate search, is widely utilized in large-scale medical clinical texts annotation and indexing. However, due to the existence of many variants of medical terminology, it is difficult for a single dictionary to cover all of them. So, it is easy to miss the undefined entity in the dictionary. In view of this issue, the more popular methods are fuzzy dictionary matching method and postprocessing method. In addition, dictionary-based approaches can intrinsically provide ID information since they recognize a term by searching the most similar (or identical) one in the dictionary to the target term. This advantage makes dictionary-based approaches particularly useful as the first step for practical information extraction from medical literature.

Bioentity name recognition is the key step for information extraction from biomedical literature. Yang et al. [14] presented a dictionary-based bioentity name recognition approach, including three processing steps: the construction and expansion of the bioentity name dictionary, the approximate string matching, and the postprocessing. The proposed approach expands the bioentity name dictionary via the abbreviation definition-identifying algorithm, improves the recall rate through the improved edit distance algorithm, and adopts some postprocessing methods. They conducted experiments using the JNLPBA2004 dataset. With these methods and based on only an internal dictionary, we achieved an F-score of 68.80% which is much better than the baseline provided by JNLPBA2004 (exact matching, 47.7%) and close to the result of the best system (72.6%).

#### 4.1.3. The Machine Learning NER Approach

Machine learning-based NER methods have shown good performance in recognizing entities in clinical text. Algorithms and features are two important factors that largely affect the performance of ML-based NER systems. The appropriate machine learning algorithm is utilized to establish the entity recognition model using the statistical characteristics and parameters of the sample data. The machine learning approach, which is data-driven and application-oriented, requires standard annotations training dataset. Various machine learning methods, such as hidden Markov models (HMM), support vector machines (SVM), conditional random field (CRF) [15], and maximum entropy (ME), are available according to data characteristics. Among a variety of machine learning algorithms, CRF methods are more popular because they allow for the incorporation of various features that can be advantageous for the process of sequence labeling.

In clinical information extraction, there are three key steps: (1) extraction of medical problems, tests, and treatments, from discharge summaries and progress notes; (2) classification of assertions made on the medical problems; and (3) classification of relations between medical concepts. de Bruijn et al. [16] developed systems, built around a (semi-) supervised machine learning paradigm, for each of the three tasks within the challenge. The systems ranked high among all submitted systems in the competition, with the following F-scores: concept extraction 0.8523 (ranked second), assertion detection 0.9362 (ranked first), and relationship detection 0.7313 (ranked second).

Tang et al. [17] investigated the use of structural support vector machines (SSVMs), an algorithm that combines the advantages of both CRFs and SVMs, and word representation features, which contain word-level backoff information over large unlabelled corpus by unsupervised algorithms, for clinical entity recognition. To compare the performance of CRFs and SSVM-based NER classifiers with the same feature sets, they used the dataset from the concept extraction task in the 2010 i2b2 NLP challenge. Evaluation results showed that the SSVM-based NER systems achieved better performance than the CRF-based systems for clinical entity recognition, when same features were used. By combining two different types of word representation features together with SSVMs, the proposed system achieved the highest *F*-measure of 85.82%, and the result outperformed the best system reported in the challenge by 0.6%, which shows that SSVM is a great potential algorithm for clinical NLP research.

To make a precise and legitimate assessment of patient discharge summaries, a proper time layout of the sequence of relevant events should be compiled and used to drive a patient-specific timeline, which could further assist medical personnel in making clinical decisions. Chang et al. [18] proposed a hybrid method to identify appropriate temporal links between a pair of entities, which combines two approaches: one is rule-based and the other is based on the maximum entropy model. The experiment results show that the proposed system achieved an F-score of 0.563, which was at least 30% better than that of the baseline system.

As part of the i2b2 2012 Natural Language Processing for Clinical Data challenge, Kovačević et al. [19] presented a system, which combines rule-based and machine learning approaches, to automatically extract temporal expressions and events from clinical narratives. Rule-based components were designed to handle the recognition and normalization of temporal expressions, while conditional random field models were trained for event and temporal recognition. The system achieved micro F-scores of 90% for the extraction of temporal expressions and 87% for clinical event extraction.

To develop and evaluate machine learning-based approaches in extracting clinical entities, Jiang et al. [20] used two ML algorithms: CRF and SVM, which have been widely applied in NER. They evaluated ML-based NER approaches using the training dataset containing 349 annotated clinical notes. In their experiments, CRF outperformed SVM with equivalent features. Additional feature and kernel optimization for the SVM may improve its performance. However, it also indicates the complexity of SVM parameter optimization. Based on the results from training data, they developed a novel hybrid clinical entity extraction system, which integrated heuristic rule-based modules with the ML-based named-entity recognition module. In the 2010 i2b2/VA NLP challenge, our system achieved a maximum F-score of 0.8391 for concept extraction (ranked second) and 0.9313 for assertion classification.

### 4.2. Relation Extraction (RE)

When the named entity is identified, the next task is to extract entity relation. According to the I2B2 2010 evaluation conference [21], the entity relations in EMR can be divided into three categories, including the relation between diseases, the relation between diseases and medical examinations, and the relation between diseases and treatment. In addition, the entity relation is limited to the relation between two named entities within a sentence.

In the medical field, three common methods are applied to extract entity relation, including cooccurrence-based [22], pattern-based, and machine learning approaches. When two entities appear in the same sentence, there is a correlation between the two entities. The higher the frequency of cooccurrence, the stronger the relation. The most widely used method is the machine learning approach. In addition, the hybrid system of two or more approaches is also developed gradually. For example, in order to handle more complex sentence structures and achieve better performance, a machine learning system based on a knowledge base or feature dictionary would be proposed.

Lee et al. [23] presented a system based on a convolutional neural network to extract relations between scientific concepts such as synonyms and hyponyms. Their model for relation extraction comprises three parts: prepocessing, convolution neural network (CNN), and rule-based postprocessing. The experiments were carried out on the ScienceIE dataset, which consists of 500 journal articles. The result is shown in Table 1.

Automatically detecting temporal relations among dates/times and events mentioned in patient records has much potential to help medical staff in understanding disease progression and patient's response to treatments. It can also facilitate evidence-based medicine (EBM) research. Yang et al. [24] proposed a hybrid temporal relation extraction approach which combines patient-record-specific rules and the conditional random fields (CRFs) model to process patient records. They evaluate the approach on the i2b2 dataset, and the results show that the proposed approach achieves an F-score of 61%.

**Figure 4:**
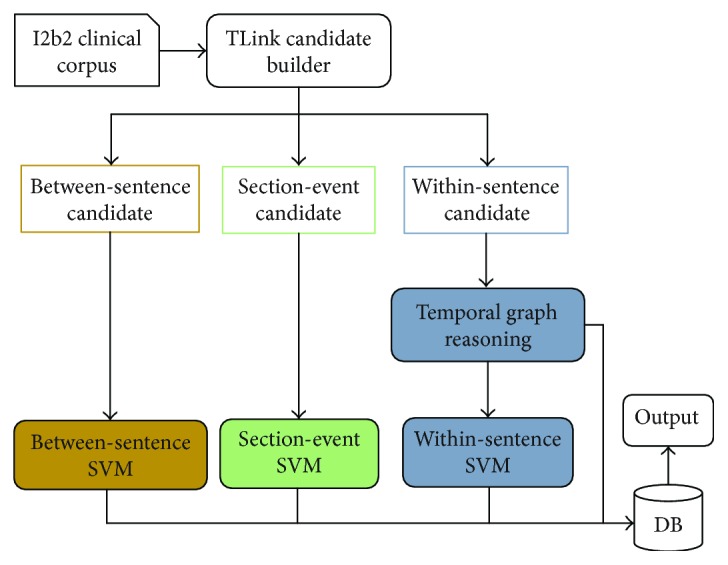
Modules of proposed system: between-sentence, sectime-event, and within-sentence.

To extract the temporal relations between pairs of events or time expressions presented in the clinical notes, Nikfarjam and Gonzalez [25] designed separate extraction components for different types of temporal relations, which utilized machine learning- and graph-based inference. The overall approach is illustrated in Figure 4. They first generated all possible TLinks and calculated the features that characterize them in the TLink Candidate Builder module. The TLinks were divided into three categories: section time-event (sectime-event), within-sentence links, and between-sentence links. For each type of TLink, a different classification pipeline was used. They trained an SVM classifier for the classification of the sectime-event candidates. The within-sentence candidates were first passed to the temporal graph reasoning module. If the type of candidate could not be determined, it was passed into the within-sentence SVM classification module. Between-sentence candidates were processed solely with a set of heuristic rules. The proposed hybrid system performance reached an *F*-measure of 0.63, with precision at 0.76 and recall at 0.54.

A chronological view of a patient's history makes clinical audits easier and improves quality of care. Seol et al. [26] proposed a method to extract clinical events related to a patient using conditional random fields and to extract relationships between events using support vector machines and to extract event causality pattern list in a semantic unit of events. The categories of events extracted are symptom, diagnosis, drug, treatment, purpose, test, finding, time, and visit. The categories of the relationships between the events are TAP, problem, action, and problem-action. The extraction of event relationships is effective, as the average of F1 is 78.8% when extracting the four types of problem-action relations.

Unstructured EHR data in the form of clinical notes can be processed to help doctors describe and reason around suspected adverse drug events (ADEs). For the purpose of learning to identify information pertaining to ADEs present in clinical notes, Henriksson et al. [27] leveraged models of distributional semantics, that is, unsupervised methods that exploit cooccurrence information to model, typically in vector space, the meaning of words and, in particular, combinations of such models, to improve the predictive performance. In their proposal, CRF was applied to generate NER models, and the random forest learning algorithm was used to label attributes of the recognized entities (negation, speculation, and temporality) and extract relations between them (indication, adverse drug event). As these tasks were tackled within the paradigm of supervised machine learning, they created a human-annotated resource of Swedish clinical notes.

## 5. Application of EMR Mining Technology

Knowledge in EMR databases can be discovered using different data mining technologies [28]. In general, there are three main areas of application.

### 5.1. Medical Decision Support and Disease Risk Prediction

It takes a lot of effort to keep doctors in possession of everything about the patients' treatment. Although medical experts will do their best to provide diagnosis and treatment, the suggestion is still based on the subjective judgment of clinical experience, and misdiagnosis and missed diagnosis are therefore likely to appear. Medical decision support systems allow medical experts to gain advice on the treatment plan of the symptoms, which is based on factual data. If the mechanism can be further developed and applied, it will play an important role in medical experts' diagnosis of diseases, especially for doctors who have less clinical experience.

Medical decision support system (MDSS) is rapidly applied, and some systems, such as the Archimedes IndiGo system, the Auminence system, the Micromedex system developed by Thomson Reuters, the Zynx medical system developed by Scott Weingarten, and the DXplain system [29] developed by the Massachusetts General Hospital (Boston), have been put into use. Take the DXplain system as an example. DXplain has been widely used since its inception in 1986, and the system is still widely used in US hospitals until now. DXplain uses a pseudoprobability algorithm to generate a sequence of diseases by inputting patient signs, disease symptoms, laboratory results, and clinical treatment. In the experiment for the accuracy of DXplain diagnosis [30], DXplain's performance was affirmed. DXplains knowledge system has expanded from the initial approximately 500 diseases to 2400 now, and there are 5000 new clinical findings and 230,000 data points.

In China, the research on medical decision support systems also has preliminary development [31, 32], which have been applied in a small range. For example, Handan Central Hospital has deployed medical decision support systems in more than 70 departments in both the East and West districts [33]. However, most medical institutes are still improving their hospital information system, and the promotion of medical decision support system is still in the theoretical and experimental research stage.

Besides, risk prediction models can also be constructed that assist doctors to judge the possibility of disease deterioration or improvement and provide better healthcare for patients with limited medical resources. Furthermore, patients can also reasonably purchase medical insurances to reduce medical costs.

### 5.2. Mobile Health, Network Medical Treatment, and Personalized Healthcare

Relying on the support of the government and the commercial operation, the mobile health system [34] takes EMR system as the core, based on medical facts rather than experience. Mobile health and network medical treatment can greatly simplify the work of hospital staff and make it more convenient to seek medical advice and more accurate to grasp physical quality.

In addition, for the concern about health, people are increasingly interested to participate in their own medical decision-making. Personalized healthcare will take into account the views of patients and formulate treatment plans and nursing methods, more in line with the actual situation of patients, such as personalized nutrition catering.

### 5.3. Disease Evolution Prediction and Drug Reaction Detection

Traditional disease and drug knowledge discovery cost a huge space-time price. However, the medical data mining technology can quickly find out the medical trajectory of the disease over time and study its natural history, with the auxiliary role for disease diagnosis and treatment. For example, in some areas with high incidence of epidemic diseases, the risk factors can be accurately identified by medical data mining technologies. In addition, after the development of new drugs, considerable funds and energy would be invested to study their effects, but the medical data mining technology can detect adverse drug events in a cost-effective way [35].

## 6. Conclusion

On the development of the Chinese EMR data processing, we believe that the following aspects need attention.

### 6.1. Public Annotated Corpus

Under the government regulations, the quantity and quality of EMR are gradually improved. However, the lake of sufficient public annotated corpus that results in the lack of the Chinese EMR processing tools and clear research tasks is the biggest obstacle to the Chinese EMR study. Therefore, the establishment of a set of hierarchical and complete Chinese public corpus is imperative. The English corpus study is more mature and systematic so that we can learn their technical implementation methods.

### 6.2. Professional Dictionary and Knowledge Base

The truth is that the study of medical dictionary and knowledge base is far behind other professional fields. Many institutes have published their own medical dictionaries, but the little useful content cannot meet the application requirements. In addition, the dictionary quality needs more appraisal and certification of specialized agencies. So, the standardization of dictionary in the medical field is worthy of attention.

### 6.3. Privacy Protection

With the deepening of EMR research, the communication between hospitals and research institutes will increase in the future and data transmission of EMR is bound to be more frequent, so more emphasis should be attached to the protection of personal privacy in EMR. However, the current simple methods, such as anonymization or security protocols, cannot meet the market demand, which needs a more manageable data protection system.

### 6.4. Reasonable Selection of Processing Tools

Processing tools should be selected according to the characteristics of EMR data and the principles of dataset design. The designed method which is of great performance in general contexts may appear performance variation in the biomedical field. We used the similarity modeling algorithm Word2vec and the word segmentation tool Ansj, to deal with pneumonia data. This method eventually achieved an accuracy rate of 25%, which presented a very poor result.

In the future, the larger scale and more complex structure of EMR will make it harder to process data in EMR, but the social and economic benefits it brings will be more remarkable and the EMR research will play a greater role in the medical field.

## Figures and Tables

**Table 1:** Result on the test set of the ScienceIE dataset, using the official train/dev/test split.

Relation	Precision	Recall	F1-score
Synonym of	0.820	0.813	0.816
Hyponym of	0.455	0.421	0.437
Microaveraged	0.658	0.633	0.645
